# Prognostic Value of a Coronary Computed Tomography Angiography–Derived Ischemia Algorithm: Comparison Against Hybrid Coronary Computed Tomography Angiography/Positron Emission Tomography Imaging

**DOI:** 10.1161/JAHA.124.040726

**Published:** 2025-11-06

**Authors:** Teemu Maaniitty, Sarah Bär, Takeru Nabeta, Jeroen J. Bax, Antti Saraste, Juhani Knuuti

**Affiliations:** ^1^ Turku PET Centre Turku University Hospital and University of Turku Turku Finland; ^2^ Department of Clinical Physiology, Nuclear Medicine and PET Turku University Hospital Turku Finland; ^3^ Department of Cardiology Bern University Hospital Inselspital Bern Switzerland; ^4^ Department of Cardiology Leiden University Medical Center Leiden the Netherlands; ^5^ Heart Center Turku University Hospital and University of Turku Turku Finland; ^6^ Faculty of Medicine University of Turku Turku Finland

**Keywords:** artificial intelligence, coronary computed tomography angiography, myocardial perfusion imaging, positron emission tomography, prognosis, Computerized Tomography (CT), Imaging, Nuclear Cardiology and PET, Prognosis

## Abstract

**Background:**

Artificial intelligence–guided quantitative computed tomography ischemia (AI‐QCT_ischemia_) is a novel machine‐learning method for predicting myocardial ischemia from coronary computed tomography angiography (CCTA). This observational cohort study aimed to compare the long‐term prognostic value of AI‐QCT_ischemia_ with hybrid CCTA/positron emission tomography (PET) myocardial perfusion imaging in suspected coronary artery disease (CAD).

**Methods:**

Symptomatic patients with suspected CAD underwent CCTA with selective downstream PET to detect ischemic CAD. Blinded reanalysis of CCTA images was done using the AI‐QCT_ischemia_ algorithm, providing a binary result (normal versus abnormal).

**Results:**

In the full analysis set (n=2271), hybrid CCTA/PET imaging was successful in 94% of the patients and AI‐QCT_ischemia_ evaluation was feasible in 83%, resulting in a per‐protocol set of 1772 patients (19% with ischemic CAD on hybrid CCTA/PET and 25% with abnormal AI‐QCT_ischemia_). There was moderate‐to‐substantial agreement between the methods (Cohen’s κ=0.61). During a median follow‐up of 7.0 years, 177 (10%) patients experienced the composite end point of all‐cause death, myocardial infarction, or unstable angina. Ischemic CAD on hybrid CCTA/PET was predictive of the composite end point (hazard ratio [HR], 2.35 [95% CI, 1.62–3.40]; *P*<0.001), after adjustment for clinical variables and early (6‐month) myocardial revascularization. Similarly, an abnormal (ischemic) AI‐QCT_ischemia_ result was independently predictive of adverse outcomes (adjusted HR, 1.98 [95% CI, 1.39–2.80]; *P*<0.001). The adjusted models, including either hybrid CCTA/PET or AI‐QCT_ischemia_, demonstrated similar discriminative ability (C‐index 0.734 versus 0.729; *P*=0.53).

**Conclusions:**

The AI‐QCT_ischemia_ algorithm demonstrated long‐term prognostic value comparable to hybrid CCTA/PET perfusion imaging in suspected CAD.

## Graphical abstract

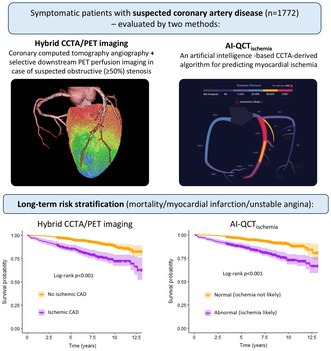

**Nonstandard Abbreviations and Acronyms**
AI‐QCT: artificial intelligence‐guided quantitative computed tomography
AI‐QCT_ischemia_
: artificial intelligence–guided quantitative computed tomography ischemia
FFR_CT_
: coronary computed tomography angiography–derived fractional flow reserve
PET: positron emission tomography
SCOT‐HEART: Scottish Computed Tomography of the Heart
UAP: unstable angina pectoris

**Clinical Perspective**

**What Is New?**

- Artificial intelligence–guided quantitative computed tomography ischemia evaluation was feasible in 83% of real‐world patients with suspected chronic coronary artery disease.
- Artificial intelligence–guided quantitative computed tomography ischemia showed moderate to substantial agreement with hybrid coronary computed tomography angiography/positron emission tomography perfusion in detecting ischemic coronary artery disease.

**What Are the Clinical Implications?**

- The long‐term prognostic value of artificial intelligence–guided quantitative computed tomography ischemia was comparable to hybrid computed tomography angiography/positron emission tomography perfusion during a median follow‐up of 7.0 years.

Myocardial perfusion imaging (MPI) has long been used in the evaluation of myocardial ischemia caused by coronary artery disease (CAD) and provides prognostic information for risk stratification.^1^, ^2^ At present, coronary computed tomography angiography (CCTA) is often the first choice for evaluation of patients with chronic chest pain, being accurate at detecting or excluding coronary atherosclerosis, while functional imaging modalities are still needed to detect myocardial ischemia.^1^, ^2^ We have previously presented a selective hybrid imaging protocol, where CCTA is used as a first‐line test, and patients with suspected obstructive stenosis on CCTA routinely undergo downstream positron emission tomography (PET) MPI for functional assessment of the stenosis. This protocol permits risk stratification but also acts as a gatekeeper to invasive coronary angiography.^3^, ^4^

More recently, CCTA‐based strategies have been evaluated for the assessment of the hemodynamic significance of coronary artery stenosis. The most extensively studied one is the CCTA‐derived fractional flow reserve (FFR_CT_) that is based on computational fluid dynamics modeling.^5^, ^6^, ^7^, ^8^ A fundamentally different CCTA‐derived approach is an artificial intelligence‐based ischemia algorithm (AI‐QCT_ischemia_) that is an Food and Drug Administration–cleared machine learning method leveraging coronary atherosclerosis and vascular morphology from CCTA images to predict the presence of hemodynamically significant coronary stenosis. An external validation study demonstrated that the diagnostic accuracy of AI‐QCT_ischemia_ is comparable to PET MPI in detecting hemodynamically significant coronary stenosis defined by invasive FFR and also suggested feasibility for risk stratification.^9^

The aim of this observational investigator‐initiated cohort study is to compare the prognostic value of the AI‐QCT_ischemia_ algorithm with hybrid CCTA/PET imaging in a real‐world patient cohort evaluated for suspected chronic CAD.

## Methods

### Patient Cohort

A total of 2411 consecutive symptomatic patients who underwent CCTA for suspected chronic CAD at Turku University Hospital, Turku, Finland, between February 2007 and December 2016 were identified from a retrospective institutional registry. Patients with known CAD or prior myocardial revascularization as well as patients referred to CCTA due to cardiomyopathy or heart failure were not included. Of the 2411 patients, 137 patients were excluded due to nonretrievable CCTA image data and 3 patients due to incomplete follow‐up. Hence, the full analysis set of this study consists of 2271 patients.

### Hybrid CCTA/PET Imaging Protocol

According to local clinical routine, all patients underwent CCTA to detect or exclude obstructive coronary stenosis.^3^ Patients with suspected obstructive (≥50% diameter) stenosis on visual CCTA reading underwent downstream PET MPI for hemodynamic evaluation of the stenosis.

Briefly, CCTA and PET scans were performed using 64‐row hybrid PET/CT scanners (GE Discovery VCT or GE D690, General Electric Medical Systems, Waukesha, WI), usually at the same imaging session. Before CCTA, all patients were administered short‐acting nitrates and, if needed, intravenous metoprolol (0–30 mg) to achieve a heart rate of <60 beats/min. Low‐osmolal iodine contrast agents were used, and prospectively electrocardiography‐triggered CCTA acquisition applied when feasible. Dynamic stress‐only PET MPI was performed with [^15^O]H_2_O tracer during adenosine vasodilator stress (140 μg/kg per min).

### Analysis and Interpretation of Hybrid CCTA/PET Imaging

A diameter stenosis ≥50% was considered obstructive on the visual reading of CCTA.^10^ Dynamic PET data were analyzed using the Carimas software (Turku PET Centre, Turku, Finland) with a standard 17 myocardial segments model.^11^ Absolute stress myocardial blood flow <2.3 mL/g per min in at least 2 adjacent segments (excluding basal septum) was considered abnormal.^12^ The results were available for the referring physicians.

Ischemic CAD by hybrid CCTA/PET imaging was defined as the concomitant presence of obstructive coronary artery stenosis on CCTA and abnormal myocardial perfusion on subsequent PET. Patients in whom ischemic CAD could not be ruled in or ruled out due to nondiagnostic image quality or who did not adhere to the selective hybrid imaging protocol (PET not performed despite obstructive stenosis on CCTA) were excluded from the per‐protocol set. In an intention‐to‐diagnose approach, these patients were considered as abnormal (ie, having ischemic CAD).

### AI‐Based CCTA‐Derived Ischemia Algorithm

The CCTA scans were reanalyzed blinded to clinical reading of CCTA/PET and patient outcomes using a previously described artificial intelligence‐guided quantitative computed tomography (AI‐QCT) algorithm (Cleerly LABS, Cleerly Inc., Denver, CO), which is a commercially available Food and Drug Administration–cleared software using convolutional neural networks.^13^, ^14^ A total of 37 output variables from AI‐QCT, for example, related to stenosis degree and plaque volumes,^9^ were subsequently imported into the Food and Drug Administration–cleared AI‐QCT_ischemia_ algorithm that determines the probability of myocardial ischemia based on CCTA variables using a random forest machine learning algorithm (Cleerly ISCHEMIA, Cleerly Inc., Denver, CO). The AI‐QCT_ischemia_ algorithm has been trained against invasive fractional flow reserve using a cutoff value of 0.80 and was externally validated in the CREDENCE (Computed Tomographic Evaluation of Atherosclerotic Determinants of Myocardial Ischemia) and PACIFIC‐1 (Comparison of Coronary Computed Tomography Angiography, Single Photon Emission Computed Tomography, Positron Emission Tomography, and Hybrid Imaging for Diagnosis of Ischemic Heart Disease Determined by Fractional Flow Reserve) study cohorts.^9^

The AI‐QCT_ischemia_ algorithm provides a binary output result: abnormal (ischemia likely) versus normal (ischemia not likely), corresponding to invasive fractional flow reserve values ≤0.80 and >0.80, respectively. The vessel‐level results consisting of the main coronary arteries as well as the side branches were first integrated at the coronary territory level (left anterior descending, left circumflex, right coronary artery) and subsequently at patient level. The per‐patient AI‐QCT_ischemia_ result was considered inconclusive if there was at least 1 nonevaluable coronary territory in the absence of any abnormal (ischemia likely) territories. A coronary territory is nonevaluable by AI‐QCT_ischemia_ if the image quality is poor, a stent is present, or the vessel is anomalous. Patients with inconclusive AI‐QCT_ischemia_ result were excluded from the per‐protocol set and considered abnormal (ischemia likely) in the intention‐to‐diagnose analysis. The results of AI‐QCT_ischemia_ were not available to referring physicians due to the retrospective nature of the assessment.

### Clinical End Points

Early myocardial revascularization was defined as percutaneous coronary intervention or coronary artery bypass grafting within 6 months after the CCTA/PET. End points included all‐cause death, myocardial infarction (MI) and unstable angina pectoris (UAP) until May 2020 derived from the registry data (Auria Clinical Informatics) and manually confirmed by investigators.

The study complies with the Declaration of Helsinki. The ethics committee of the Hospital District of Southwest Finland approved the study protocol and waived the need for written informed consent due to the observational study design. The data supporting the findings are not publicly available due to privacy and ethical restrictions.

### Statistical Analysis

Continuous variables are presented as median (interquartile range) and compared using the Mann–Whitney *U* test (nonnormal distribution based on the Shapiro–Wilk test). Categorical variables are expressed as numbers (percentages) and compared using the χ^2^ test. Cohen’s κ was calculated as a measure of agreement. The primary end point was the composite of all‐cause death, MI, or UAP, whichever occurred first. Furthermore, analyses were performed for different event types separately. Annual rate (percentage) of the composite end point with 95% Wald CIs was calculated as number of adverse events divided by patient‐years of follow‐up. Kaplan–Meier curves with 95% CIs were fitted and compared with the log‐rank test. Univariable and multivariable Cox regression analyses were performed and hazard ratios (HRs) reported with 95% CIs based on robust standard error estimators. A CI including unity (ie, 1) does not rule out either a positive or a negative (potentially clinically relevant) effect. As a predefined approach, clinical variables showing significant univariable associations and early (6‐month) myocardial revascularization were included in multivariable Cox regression models. However, acknowledging the potential limitations of different variable selection processes,^15^ we also tested fully adjusted multivariable models (not shown) and created a directed acyclic graph showing assumed relationships between our study variables and suggesting that our multivariable Cox regression models sufficiently address for confounding (Figure S1). The receiver operating characteristic curves were plotted for multivariable Cox models. Harrell’s C‐index with corresponding 95% CI was calculated for different multivariable Cox models and compared using a method designed for 2 correlated C‐indices (R package compareC). Two‐tailed *P*‐value <0.05 was considered statistically significant. Statistical analyses were conducted using IBM SPSS Statistics for Windows, version 27.0 (IBM Corp., Armonk, NY) and R (R Core Team, 2021; R Foundation for Statistical Computing, Vienna, Austria) with packages survival, survminer, pROC, survcomp, and compareC.

## Results

### Feasibility of Evaluation

**Figure 1:**
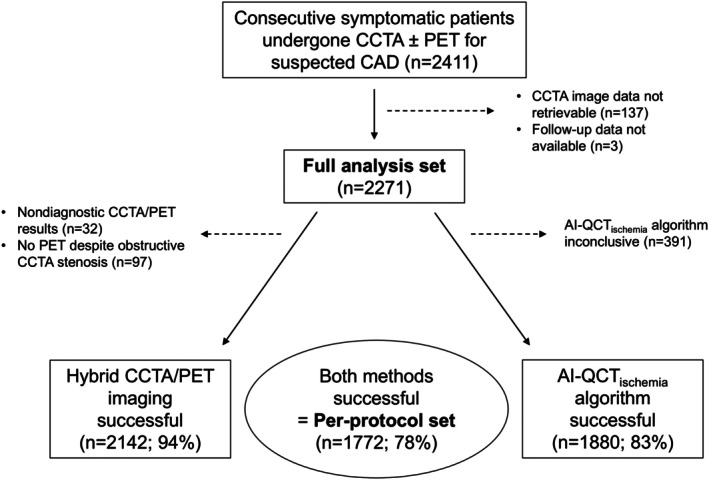
Study flowchart. Patients who both successfully completed the selective hybrid CCTA/PET protocol and had conclusive results by AI‐QCT_ischemia_ algorithm constitute the per‐protocol set of this study. AI‐QCT_ischemia_ indicates artificial intelligence‐guided quantitative computed tomography ischemia; CAD, coronary artery disease; CCTA, coronary computed tomography angiography; and PET, positron emission tomography.

As shown in the study flowchart (Figure 1), of the 2271 patients in the full analysis set, 2142 (94%) successfully completed the hybrid CCTA/PET imaging protocol and were included in the per‐protocol set, whereas 32 (1.4%) with nondiagnostic CCTA/PET and 97 (4.3%) who did not undergo PET despite obstructive CAD on CCTA were excluded.

Of the 2271 patients in the full analysis set, 1880 (83%) patients underwent successful evaluation using the AI‐QCT_ischemia_ algorithm, whereas 391 (17%) patients had inconclusive results and were excluded from the per‐protocol set (Figure 1). At the territory level, the AI‐QCT_ischemia_ evaluation was successful in 6086 (89%) of the 6813 coronary territories (left anterior descending, 2065/2271; left circumflex, 2040/2271; and right coronary artery, 1981/2271).

A total of 1772 patients (median age, 63 years; 44% men) who underwent successful evaluation by both hybrid CCTA/PET imaging and the AI‐QCT_ischemia_ algorithm constitute the per‐protocol set of this study (Figure 1). Characteristics of patients in the per‐protocol set are shown in Table 1.

**Table 1:** Patient Characteristics in Total Cohort (Per‐Protocol Set) and According to Hybrid CCTA/PET Imaging and AI‐QCT_ischemia_ Algorithm

Per‐protocol set	Total	Hybrid CCTA/PET imaging			AI‐QCT_ischemia_ algorithm		
		No ischemic CAD	Ischemic CAD	*P* value	Normal result	Abnormal result	*P* value
	n=1772	n=1441	n=331		n=1325	n=447	
Age, y	63 (56–69)	62 (55–69)	66 (59–71)	<0.001	62 (55–68)	67 (61–72)	<0.001
Male sex	776	548	228	<0.001	503	273	<0.001
	(44)	(38)	(69)		(38)	(61)	
Smoking (current or previous)	589	442	147	<0.001	406	183	<0.001
	(33)	(31)	(44)		(31)	(41)	
Diabetes	262	192	70	<0.001	164	98	<0.001
	(15)	(13)	(21)		(12)	(22)	
Hypertension	988	764	224	<0.001	677	311	<0.001
	(56)	(53)	(68)		(51)	(70)	
Dyslipidemia	1147	898	249	<0.001	821	326	<0.001
	(65)	(62)	(75)		(62)	(73)	
Family history of CAD	845	693	152	0.476	642	203	0.266
	(48)	(48)	(46)		(48)	(45)	
Typical angina	405	295	110	<0.001	269	136	<0.001
	(23)	(20)	(33)		(20)	(30)	
Medications							
Statin or ezetimibe	799	597	202	<0.001	530	269	<0.001
	(45)	(41)	(61)		(40)	(60)	
ACE inhibitor or ATR blocker	732	560	172	<0.001	508	224	<0.001
	(41)	(39)	(52)		(38)	(50)	
Antiplatelet drug	780	588	192	<0.001	529	251	<0.001
	(44)	(41)	(58)		(40)	(56)	
Anticoagulant	146	119	27	0.952	103	43	0.220
	(8)	(8)	(8)		(8)	(10)	
β blocker	865	661	204	<0.001	591	274	<0.001
	(49)	(46)	(62)		(45)	(61)	
Calcium channel blocker	296	226	70	0.016	191	105	<0.001
	(17)	(16)	(21)		(14)	(23)	
Hybrid CCTA/PET imaging findings				<0.001			<0.001
No CAD	543	543	0		542	1	
	(31)	(38)	(0)		(41)	(0)	
Nonobstructive CAD	567	567	0		537	30	
	(32)	(39)	(0)		(41)	(7)	
Obstructive CAD without ischemia	331	331	0		184	147	
	(19)	(23)	(0)		(14)	(33)	
Obstructive CAD with ischemia	331	0	331		62	269	
	(19)	(0)	(100)		(5)	(60)	
Early (6‐mo) revascularizations							
Early PCI	139	21	118	<0.001	14	125	<0.001
	(8)	(1)	(36)		(1)	(28)	
Early CABG	33	0	33	<0.001	1	32	<0.001
	(2)	(0)	(10)		(0)	(7)	
Early PCI or CABG	169	21	148	<0.001	15	154	<0.001
	(10)	(1)	(45)		(1)	(34)	
Cumulative adverse events							
Number of deaths	116	71	45	<0.001	67	49	<0.001
	(7)	(5)	(14)		(5)	(11)	
Number of MIs	49	27	22	<0.001	15	34	<0.001
	(3)	(2)	(7)		(1)	(8)	
Number of UAPs	27	10	17	<0.001	6	21	<0.001
	(2)	(1)	(5)		(0)	(5)	
Composite end point (death/MI/UAP)	177	103	74	<0.001	87	90	<0.001
	(10)	(7)	(22)		(7)	(20)	

Values are reported as median (interquartile range) and n (%). Age was compared between groups using the Mann–Whitney *U* test (nonnormal distribution). Categorical variables were compared using the χ2 test. ACE indicates angiotensin‐converting enzyme, AI‐QCT_ischemia_, artificial intelligence‐guided quantitative computed tomography ischemia; ATR, angiotensin receptor; CABG, coronary artery bypass grafting; CAD, coronary artery disease; CCTA, coronary computed tomography angiography; MI, myocardial infarction; PCI, percutaneous coronary intervention; PET, positron emission tomography; and UAP, unstable angina pectoris.

### Imaging Findings

**Figure 2:**
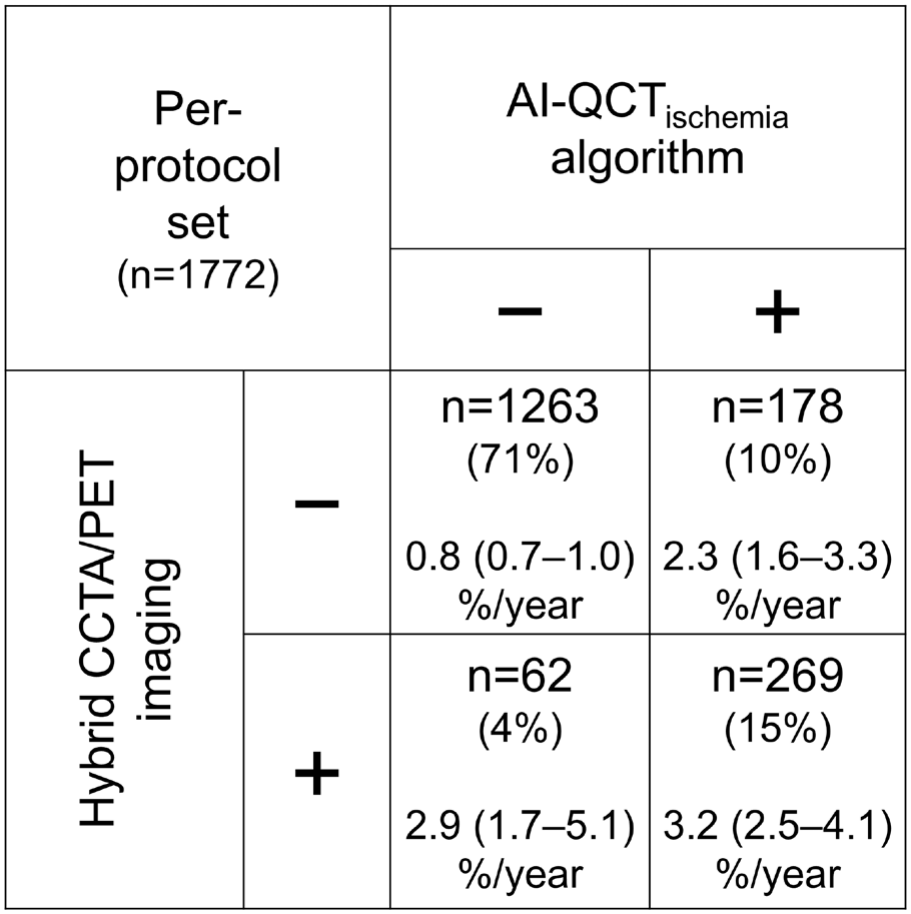
Distribution of imaging findings in per‐protocol set. The number (percentage) of patients is shown according to the absence (−) or presence (+) of ischemic CAD by hybrid CCTA/PET imaging and normal (−) or abnormal (+) result by AI‐QCT_ischemia_ algorithm. In addition, the annual rate of the composite end point (death/MI/UAP) with 95% confidence intervals is shown for each subgroup. AI‐QCT_ischemia_ indicates artificial intelligence‐guided quantitative computed tomography ischemia; CAD, coronary artery disease; CCTA, coronary computed tomography angiography; MI, myocardial infarction; PET, positron emission tomography; and UAP, unstable angina pectoris.

In the per‐protocol set (n=1772), CCTA revealed no CAD in 543 (31%) patients and nonobstructive CAD in 567 (32%) patients, whereas 662 (37%) patients had obstructive CAD triggering downstream PET perfusion imaging. Subsequent PET MPI showed normal results in 331 (19% of 1772) patients, whereas 331 (19%) patients had ischemic CAD. In comparison, the AI‐QCT_ischemia_ result was abnormal in 447 (25% of 1772) patients, suggesting the presence of ischemia. Figure 2 demonstrates moderate to substantial agreement between hybrid CCTA/PET imaging and the AI‐QCT_ischemia_ algorithm (Cohen’s κ=0.61, χ^2^ test *P*<0.001). There was concordant presence of ischemia in 269 (15%) and concordant absence of ischemia in 1263 (71%) patients. Compared with the per‐protocol set, the patient‐level agreement was lower in the full analysis set (Cohen’s κ=0.39, χ^2^ test *P*<0.001), with concordant findings in 1657 (73%) of 2271 patients (Figure S2). Furthermore, the agreement on coronary territory level was moderate (Cohen’s κ=0.46, χ^2^ test *P*<0.001), with concordant ischemic findings in 355 (6%) and concordant nonischemic findings in 4748 (83%) of the 5741 territories (from 2271 patients) that had been successfully analyzed by both methods.

### Prognostic Value of Imaging Findings

**Figure 3:**
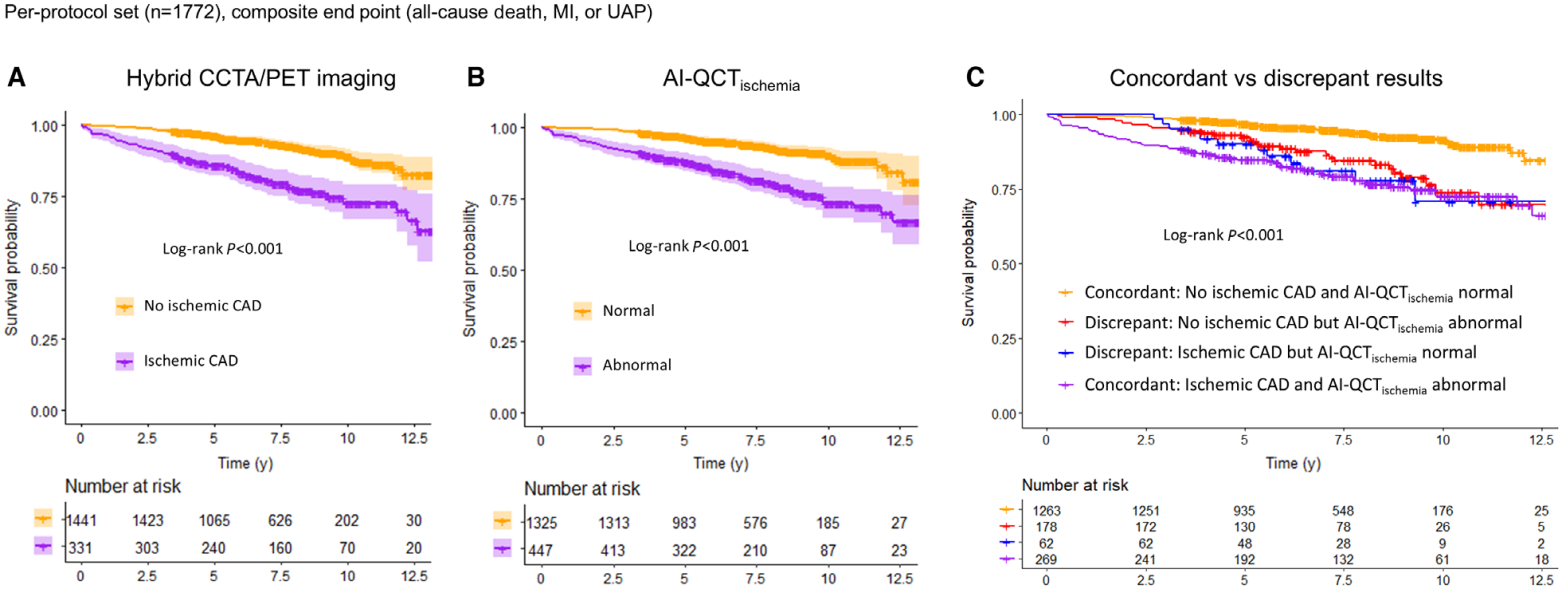
Risk stratification by imaging findings in per‐protocol set. Kaplan–Meier survival curves (with shaded 95% confidence intervals) demonstrating the discriminative power of hybrid CCTA/PET imaging (**A**) and AI‐QCT_ischemia_ algorithm (**B**) in predicting long‐term outcome (composite of all‐cause death, myocardial infarction, and unstable angina pectoris). In addition, risk stratification based on concordant or discrepant findings by hybrid CCTA/PET and AI‐QCT_ischemia_ is shown (**C**). AI‐QCT_ischemia_ indicates artificial intelligence‐guided quantitative computed tomography ischemia; CAD, coronary artery disease; CCTA, coronary computed tomography angiography; MI, myocardial infarction; PET, positron emission tomography; and UAP, unstable angina pectoris.

Median follow‐up time was 7.0 years (interquartile range, 4.9–9.1 years), during which 177 (10%) of 1772 patients experienced the composite end point of death/MI/UAP (Table 1, Figure 2). In 24 of 27 patients presenting with UAP, treatment strategy included revascularization. In the per‐protocol set, Kaplan–Meier survival curves (Figure 3) showed similar discriminative power of hybrid CCTA/PET imaging and the AI‐QCT_ischemia_ algorithm for the prediction of long‐term outcomes in terms of the composite end point (see Figure S3 for full analysis set). Survival curves for each component of the end point are shown in Figure S4. The clinical outcome of patients who had discrepant imaging findings (hybrid CCTA/PET versus AI‐QCT_ischemia_) was intermediate, approaching that of patients with concordant ischemic imaging findings toward the end of follow‐up (Figure 3). The performance rate of early revascularization was 0.3% and 51% in patients with concordant nonischemic and concordant ischemic findings, respectively. Among patients with discrepant findings, early revascularization was performed in 10% of those with an abnormal AI‐QCT_ischemia_ result and in 18% of those with ischemic CAD on hybrid CCTA/PET.

**Figure 4:**
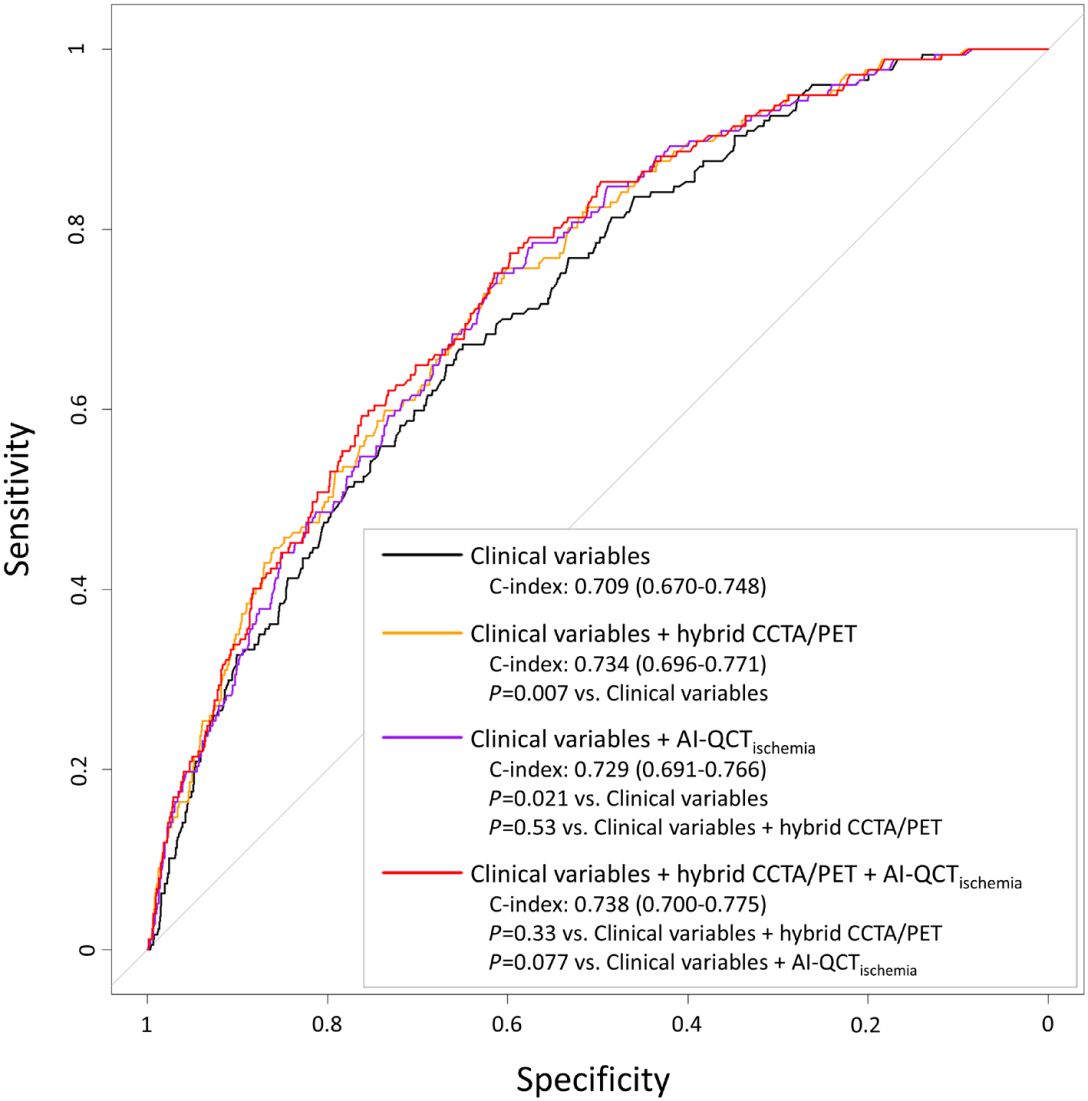
Predictive power of imaging findings in per‐protocol set. Receiver operating characteristic curves are presented for different multivariable models in predicting the composite end point (death/MI/UAP). Harrell’s C‐index with 95% confidence interval is shown for different multivariable models. AI‐QCT_ischemia_ indicates artificial intelligence‐guided quantitative computed tomography ischemia; CCTA, coronary computed tomography angiography; MI, myocardial infarction; PET, positron emission tomography; and UAP, unstable angina pectoris.

Multivariable Cox regression analysis (Table 2) demonstrates that ischemic CAD detected by hybrid CCTA/PET was an independent predictor of the composite end point (adjusted HR, 2.35 [95% CI, 1.62–3.40]; *P*<0.001), after adjustment for clinical predictors and early revascularization. Similarly, an abnormal AI‐QCT_ischemia_ result was an independent prognostic predictor when adjusted for clinical predictors and early revascularization (adjusted HR, 1.98 [95% CI, 1.39–2.80]; *P*<0.001). The discriminative ability of these 2 models was similar in terms of C‐index (0.734 [95% CI, 0.696–0.771] versus 0.729 [95% CI, 0.691–0.766], respectively; *P*=0.53), and superior to the reference model including only clinical variables (0.709 [95% CI, 0.670–0.748]; *P*=0.007 versus hybrid CCTA/PET model, and *P*=0.021 versus AI‐QCT_ischemia_ model; Figure 4).

**Table 2:** Predictors of Outcome in Per‐Protocol Set

Per‐protocol set (n=1772)	Univariable associations	*P* value	Multivariable model 1: Clinical variables	*P* value	Multivariable model 2: Hybrid CCTA/PET imaging adjusted for clinical variables and early revascularization	*P* value	Multivariable model 3: AI‐QCT_ischemia_ algorithm adjusted for clinical variables and early revascularization	*P* value	Multivariable model 4: Hybrid CCTA/PET imaging and AI‐QCT_ischemia_ algorithm adjusted for clinical variables and early revascularization	*P* value
Model C‐index (95% CI)	N/A		0.709 (0.670–0.748)		0.734 (0.696–0.771)		0.729 (0.691–0.766)		0.738 (0.700–0.775)	
Age (per 1 year)	1.07 (1.05–1.09)*	<0.001*	1.07 (1.05–1.10)*	<0.001*	1.07 (1.05–1.09)*	<0.001*	1.06 (1.04–1.09)	<0.001*	1.06 (1.04–1.09)*	<0.001*
Male sex	1.55 (1.15–2.09)*	0.004*	1.67 (1.23–2.25)*	0.001*	1.42 (1.04–1.95)*	0.029*	1.48 (1.08–2.02)	0.014*	1.38 (1.01–1.89)*	0.046*
Smoking history	1.48 (1.10–2.00)*	0.009*	1.52 (1.12–2.07)*	0.007*	1.47 (1.08–2.00)*	0.014*	1.48 (1.09–2.01)	0.012*	1.47 (1.08–2.00)*	0.014*
Diabetes	1.75 (1.23–2.50)*	0.002*	1.33 (0.92–1.92)	0.131	1.29 (0.89–1.88)	0.185	1.27 (0.87–1.83)	0.213	1.27 (0.87–1.84)	0.216
Hypertension	1.75 (1.28–2.40)*	0.001*	1.37 (0.98–1.91)	0.063	1.30 (0.93–1.82)	0.121	1.30 (0.93–1.82)	0.122	1.28 (0.92–1.79)	0.148
Dyslipidemia	1.00 (0.73–1.36)	0.993								
Family history of CAD	0.82 (0.61–1.11)	0.196								
Typical angina	1.61 (1.18–2.22)*	0.003*	1.45 (1.05–2.00)*	0.026*	1.35 (0.97–1.89)	0.078	1.37 (0.98–1.90)	0.064	1.34 (0.96–1.87)	0.085
Ischemic CAD by hybrid CCTA/PET imaging	2.98 (2.19–4.04)*	<0.001*			2.35 (1.62–3.40)*	<0.001*			1.90 (1.25–2.89)*	0.003*
Abnormal result by AI‐QCT_ischemia_ algorithm	2.96 (2.19–3.99)*	<0.001*					1.98 (1.39–2.80)*	<0.001*	1.52 (1.03–2.26)*	0.037*
Early revascularization	1.86 (1.26–2.76)*	0.002*			0.70 (0.43–1.14)	0.148	0.83 (0.53–1.31)	0.418	0.64 (0.40–1.04)	0.072

Univariable and multivariable Cox regression models show the associations of hybrid CCTA/PET imaging findings and AI‐QCT_ischemia_ algorithm with the composite end point (death/MI/UAP), adjusted for clinical variables and early revascularization. Hazard ratios with 95% CIs are presented. Harrell’s C‐index is presented for multivariable models (with 95% CI). AI‐QCT_ischemia_ indicates artificial intelligence‐guided quantitative computed tomography ischemia; CAD, coronary artery disease; CCTA, coronary computed tomography angiography; MI, myocardial infarction; N/A, not applicable; PET, positron emission tomography; and UAP, unstable angina pectoris.

* *P* <0.05.

Subsequently, both hybrid CCTA/PET and AI‐QCT_ischemia_ results were included in a multivariable Cox regression model along with clinical predictors and early revascularization, in which both ischemic CAD by hybrid CCTA/PET and abnormal AI‐QCT_ischemia_ result remained independent predictors of outcome (adjusted HR, 1.90 [95% CI 1.25–2.89], *P*=0.003, and adjusted HR, 1.52 [95% CI 1.03–2.26], *P*=0.037, respectively). The discriminative ability of this model (C‐index 0.738 [95% CI, 0.700–0.775]) was not statistically superior to the model with clinical variables along with AI‐QCT_ischemia_ (*P*=0.077) or hybrid CCTA/PET (*P*=0.33).

Intention‐to‐diagnose analysis also demonstrated comparable discriminative ability of a model with clinical variables and hybrid CCTA/PET (C‐index 0.727, 95% CI 0.696–0.759) and a model including clinical variables and AI‐QCT_ischemia_ (C‐index 0.714, 95% CI 0.683–0.745; *P*=0.12) (Table S1, Figure S5).

Multivariable Cox regression analysis for different components of the end point suggests that an abnormal AI‐QCT_ischemia_ result more specifically predicts cardiac events (ie, MI and UAP), whereas hybrid CCTA/PET predicts all‐cause death (Table S2).

## Discussion

In this observational study of real‐world symptomatic patients evaluated for suspected CAD, we compared the prognostic value of myocardial ischemia determined by 2 different approaches: a novel AI‐QCT_ischemia_ algorithm and clinical hybrid imaging with first‐line CCTA followed by selective downstream PET MPI. There was a moderate to substantial agreement between the 2 approaches, with concordant presence of ischemia in 13% and concordant absence of ischemia in 75% of patients. AI‐QCT_ischemia_ algorithm and hybrid CCTA/PET demonstrated similar prognostic value during a median follow‐up of 7.0 years.

Currently, CCTA is often the method of choice for evaluation of patients with suspected chronic CAD.^1^, ^2^ The randomized SCOT‐HEART (Scottish Computed Tomography of the Heart) trial demonstrated that the ability of CCTA to detect nonobstructive or obstructive coronary atherosclerosis is associated with increased use of preventive medication, and adding CCTA on top of standard care improves clinical outcomes.^16^ However, the anatomic degree of coronary artery stenosis on CCTA does not accurately predict myocardial ischemia, which is an important criterion when considering myocardial revascularization.^17^, ^18^ Therefore, downstream testing with functional imaging modalities may be necessary after CCTA.^1^ The selective hybrid CCTA/PET protocol used in our hospital takes advantage of the high negative predictive value of first‐line CCTA, whereas an obstructive stenosis on CCTA triggers downstream evaluation of ischemia by PET.^3^, ^12^, ^19^

In the current study using real‐world CCTA data, we found that AI‐QCT_ischemia_ evaluation was feasible in 89% of the major coronary territories, resulting in complete evaluation in 83% of the patients. For comparison, a previous intention‐to‐diagnose analysis of FFR_CT_ demonstrated successful evaluation in 83% of major coronary arteries, resulting in complete (ie, 3‐vessel) FFR_CT_ assessment in 75% of patients.^6^ Recently, an external validation study reported that 96% of major coronary arteries were interpretable using AI‐QCT_ischemia_, whereas FFR_CT_ was interpretable in 84% and MPI in 98% of the vessels.^9^ Different success rates of FFR_CT_ and AI‐QCT_ischemia_ may be related to inherent differences between these methods, the former being based on computational fluid dynamics modeling of stenosis, whereas the latter uses machine learning and 37 coronary anatomy‐related parameters.^5^

We found that an abnormal AI‐QCT_ischemia_ result (ischemia likely) provides similar long‐term risk stratification as the presence of ischemic CAD by hybrid CCTA/PET. Both methods showed ≈3‐fold unadjusted and 2‐fold adjusted rates for the composite end point (death/MI/UAP) when ischemia was present (versus nonischemic findings). In a meta‐analysis, the presence of reduced FFR_CT_ was associated with a 2.3‐fold unadjusted relative risk for the composite end point of all‐cause death or MI at short‐term (1 year) follow‐up.^20^ Another study showed that abnormal FFR_CT_ was associated with 2.5‐fold adjusted risk for all‐cause death or MI at 3‐year follow‐up.^21^

The long‐term prognostic value of AI‐QCT_ischemia_ has been previously addressed in a cohort of 204 participants in the PACIFIC‐1 trial and ≈7‐fold adjusted event risk was found for abnormal versus normal AI‐QCT_ischemia_ result, which is remarkably higher than in our study.^9^ However, it is also important to note that late myocardial revascularization was included as an end point in that study. Furthermore, when interpreting the results of our study, it should be acknowledged that those with normal (nonischemic) findings by AI‐QCT_ischemia_ and/or hybrid CCTA/PET comprise a large heterogeneous group, ranging from absence of coronary atherosclerosis to anatomically extensive or obstructive, but functionally nonsignificant CAD. Recently, we reported that AI‐QCT_ischemia_ might be particularly useful to improve risk stratification in patients without obstructive CAD on coronary CTA who are typically considered as a low‐risk group by perfusion imaging (often normal perfusion).^22^ On the other hand, the adherence of revascularization with perfusion imaging findings appears high in our center, potentially leading to elimination of ischemia and reduced adverse event rates.^4^

The current study demonstrates moderate to substantial agreement in ischemic classification based on the AI‐QCT_ischemia_ algorithm and hybrid CCTA/PET imaging. However, 14% of patients in the per‐protocol set and up to 27% in the intention‐to‐diagnose approach were discordantly classified by these 2 methods. Interestingly, both methods have been validated against invasive fractional flow reserve using a cutoff value of 0.80.^9^, ^12^, ^19^ Still, the observed discrepancies are not entirely unexpected since AI‐QCT_ischemia_ is derived from static CCTA images of epicardial coronary arteries, whereas PET perfusion is based on functional assessment of myocardial blood flow and integrates both epicardial and microvascular circulation. We have focused on the factors that might explain these discrepancies in our recently published paper.^23^ Moreover, our observation of lower agreement between AI‐QCT_ischemia_ and hybrid CCTA/PET imaging on coronary territory level as compared with patient level may be also partly related to, for example, individual variations in coronary artery anatomy. Importantly, patients with both tests indicating absence of ischemia had favorable outcomes. Furthermore, our study cohort suggested that abnormal AI‐QCT_ischemia_ may more specifically predict cardiac events (MI and UAP), whereas hybrid CCTA/PET imaging predicts all‐cause death. We can hypothesize that this could be related to the fact that AI‐QCT_ischemia_ algorithm incorporates features such as quantitated coronary atherosclerotic plaque volumes that are known to be predictors of cardiovascular adverse events.^9^, ^24^ Our study findings related to discrepancies between AI‐QCT_ischemia_ and hybrid CCTA/PET imaging as well as their time‐dependent and event‐specific prognostic value warrant further research.

### Study Limitations

The current study has inherent limitations related to the retrospective design. However, the AI‐QCT_ischemia_ analysis was performed blinded to clinical data, clinical CCTA readings, and PET findings. The patients were dichotomously classified as either having or not having ischemic CAD on the basis of hybrid CCTA/PET imaging, that was justified by the fact that AI‐QCT_ischemia_ algorithm provides only a binary result (normal versus abnormal), without an option to grade the severity of ischemia. Moreover, PET MPI was not performed in all patients but selectively used in patients with obstructive CCTA findings, aiming at detecting hemodynamically significant epicardial CAD rather than coronary microvascular dysfunction.^3^ Since the causes of death were not available, all‐cause death was used as an end point, however, thereby avoiding verification bias. We have earlier shown that the adherence of revascularization decisions to the results of hybrid CCTA/PET imaging is very high.^4^ As the CCTA/PET results were available to guide clinicians, this may have potentially impacted outcome, whereas AI‐QCT_ischemia_ was retrospectively derived by reanalysis of CCTA images. Accordingly, to account for the potential effect of revascularization, this was included as a covariate in Cox regression models. Finally, AI‐QCT_ischemia_ demonstrated higher rates of abnormal (ischemic) and inconclusive results as compared with hybrid CCTA/PET imaging, and their impact on downstream resource use (eg, unnecessary invasive coronary angiographies) warrants future prospective studies.

## Conclusions

In symptomatic patients with suspected chronic CAD, the AI‐QCT_ischemia_ algorithm provided similar long‐term prognostic value as compared with hybrid CCTA/PET perfusion imaging, for the prediction of the composite end point of all‐cause death, MI, and UAP. AI‐QCT_ischemia_ may more specifically predict cardiac events (MI and UAP), whereas hybrid CCTA/PET imaging predicts all‐cause death. However, the clinical impact of inconclusive AI‐QCT_ischemia_ results needs further investigation.

## Sources of Funding

Authors report financial support from the Research Council of Finland; the Finnish Foundation for Cardiovascular Research; Finnish State Research Funding for Turku University Hospital, the University of Turku, Finland; and the Swiss National Science Foundation. Cleerly, Inc. performed AI‐QCT_ischemia_ analysis without costs and provided an unrestricted research grant to University of Turku.

## Disclosures

Dr Bär reports research grants to the institution from Medis Medical Imaging Systems, Bangerter‐Rhyner Stiftung (Basel, Switzerland) and Abbott, outside the submitted work. Dr Saraste received consultancy fees from AstraZeneca, Novo Nordisc, and Pfizer; and speaker fees from Abbott, AstraZeneca, BMS, Janssen, and Pfizer. Dr Bax received speaker fees from Abbott. Dr Knuuti received consultancy fees from GE Healthcare and Synektik and speaker fees from Bayer, Lundbeck, Boehringer‐Ingelheim, Pfizer, and Siemens, outside of the submitted work. The remaining authors have no disclosures to report.

## Supporting information

Tables S1–S2
Figures S1–S5
