# Supplementary material for: Prognostic Value of a Coronary Computed Tomography Angiography–Derived Ischemia Algorithm: Comparison Against Hybrid Coronary Computed Tomography Angiography/Positron Emission Tomography Imaging
Source: J Am Heart Assoc. 2025 Nov 6;14(22):e040726. doi: 10.1161/JAHA.124.040726 (PMC12887235; doi:10.1161/JAHA.124.040726)
Supplement: Supplementary file 1 — Tables S1–S2 Figures S1–S5 [file JAH3-14-e040726-s001.pdf]

Supplemental Material

Table S1. Predictors of outcome in full analysis set (intention-to-diagnose approach). Univariable and multivariable Cox regression models show the associations of hybrid CCTA/PET imaging findings and AI-QCT$_{ischemia}$ algorithm with the composite endpoint (death/MI/UAP), adjusted for clinical variables and early revascularization.

**Full analysis set** n=2271	**Univariable associations**	P-value	**Multivariable model 1:** Clinical variables	P-value	**Multivariable model 2:** Hybrid CCTA/PET imaging adjusted for clinical variables and early revascularization	P-value	**Multivariable model 3:** AI-QCT$_{ischemia}$ algorithm adjusted for clinical variables and early revascularization	P-value	**Multivariable model 4:** Hybrid CCTA/PET imaging and AI-QCT$_{ischemia}$ algorithm adjusted for clinical variables and early revascularization	P-value
Model C-index (95%CI)	N/A		0.694 (0.661-0.727)		0.727 (0.696-0.759)		0.714 (0.683-0.745)		0.732 (0.701-0.763)	
Age (per 1 year)	**1.07 (1.05-1.09)**	**<0.001**	**1.07 (1.06-1.09)**	**<0.001**	**1.07 (1.05-1.09)**	**<0.001**	**1.07 (1.05-1.08)**	**<0.001**	**1.07 (1.05-1.08)**	**<0.001**
Male sex	**1.44 (1.13-1.84)**	**0.004**	**1.55 (1.20-1.99)**	**<0.001**	**1.32 (1.01-1.73)**	**0.046**	**1.45 (1.12-1.89)**	**0.005**	1.31 (1.00-1.71)	0.051
Smoking history	**1.44 (1.12-1.85)**	**0.004**	**1.52 (1.17-1.96)**	**0.001**	**1.48 (1.14-1.92)**	**0.003**	**1.48 (1.14-1.91)**	**0.003**	**1.47 (1.14-1.90)**	**0.003**
Diabetes	**1.61 (1.19-2.17)**	**0.002**	1.27 (0.93-1.73)	0.128	1.19 (0.87-1.63)	0.269	1.22 (0.90-1.66)	0.205	1.18 (0.87-1.62)	0.288
Hypertension	**1.78 (1.36-2.31)**	**<0.001**	**1.40 (1.06-1.85)**	**0.019**	1.31 (0.99-1.75)	0.058	**1.34 (1.01-1.77)**	**0.041**	1.29 (0.97-1.71)	0.079
Dyslipidemia	0.98 (0.76-1.26)	0.851								
Family history of CAD	0.84 (0.65-1.07)	0.159								
Presence of typical AP	**1.42 (1.09-1.87)**	**0.011**	**1.32 (1.01-1.74)**	**0.045**	1.23 (0.93-1.62)	0.152	1.26 (0.96-1.67)	0.097	1.22 (0.92-1.62)	0.158
Ischemic CAD by hybrid CCTA/PET imaging	**2.93 (2.28-3.76)**	**<0.001**			**2.36 (1.76-3.17)**	**<0.001**			**2.04 (1.49-2.79)**	**<0.001**
Abnormal result by AI-QCT$_{ischemia}$ algorithm	**2.47 (1.91-3.18)**	**<0.001**					**1.83 (1.39-2.41)**	**<0.001**	**1.50 (1.12-2.02)**	**0.007**
Early revascularization	**1.83 (1.31-2.56)**	**<0.001**			0.75 (0.51-1.12)	0.156	0.96 (0.66-1.41)	0.852	0.69 (0.47-1.03)	0.071

Hazard ratios with 95% confidence intervals (CI) in parentheses are presented. Harrell's C-index is presented for multivariable models (with 95%CI in parentheses).

AI-QCT = artificial intelligence -guided quantitative computed tomography, AP = angina pectoris, CAD = coronary artery disease, CCTA = coronary computed tomography angiography, MI = myocardial infarction, PET = positron emission tomography, UAP = unstable angina pectoris

Table S2. Predictors of different adverse event types. Multivariable Cox regression analysis separately for different endpoints (all-cause mortality, myocardial infarction, and unstable angina pectoris) in the per-protocol set. Due to limited number of each type of adverse events, the analyses were adjusted only for age, sex, and smoking history (based on the significant associations with the composite endpoint in the fully adjusted model).

**Per-protocol set** n=1772	Endpoint: **All-cause mortality**	P-value	Endpoint: **Myocardial infarction**	P-value	Endpoint: **Unstable angina pectoris**	P-value
Number of events	116		49		27	
Age (per 1 year)	**1.09 (1.07-1.12)**	**<0.001**	1.04 (1.00-1.08)	0.085	1.03 (0.98-1.08)	0.231
Male sex	1.30 (0.89-1.91)	0.180	1.17 (0.68-2.02)	0.574	1.04 (0.46-2.36)	0.926
Smoking history	**1.59 (1.10-2.30)**	**0.013**	1.75 (1.00-3.04)	0.049	1.30 (0.60-2.82)	0.518
Ischemic CAD by hybrid CCTA/PET imaging	**1.96 (1.14-3.36)**	**0.014**	1.03 (0.52-2.02)	0.943	2.34 (0.88-6.17)	0.072
Abnormal result by AI-$QCT_{ischemia}$ algorithm	0.82 (0.48-1.41)	0.478	**4.99 (2.41-10.33)**	**<0.001**	**4.97 (1.60-15.41)**	**0.002**

Hazard ratios (with 95% confidence intervals in parentheses) are presented.

AI-QCT = artificial intelligence -guided quantitative computed tomography, CAD = coronary artery disease, CCTA = coronary computed tomography angiography, PET = positron emission tomography.

Figure S1. A directed acyclic graph (DAG) showing assumed relationships between study variables and suggesting that the multivariable Cox regression models sufficiently address for confounding. More specifically, conditioning on a set of variables included in the multivariable models, there do not remain unblocked paths between myocardial ischemia (detected by hybrid CCTA/PET or AI-QCT$_{ischemia}$) and adverse clinical outcome (events).

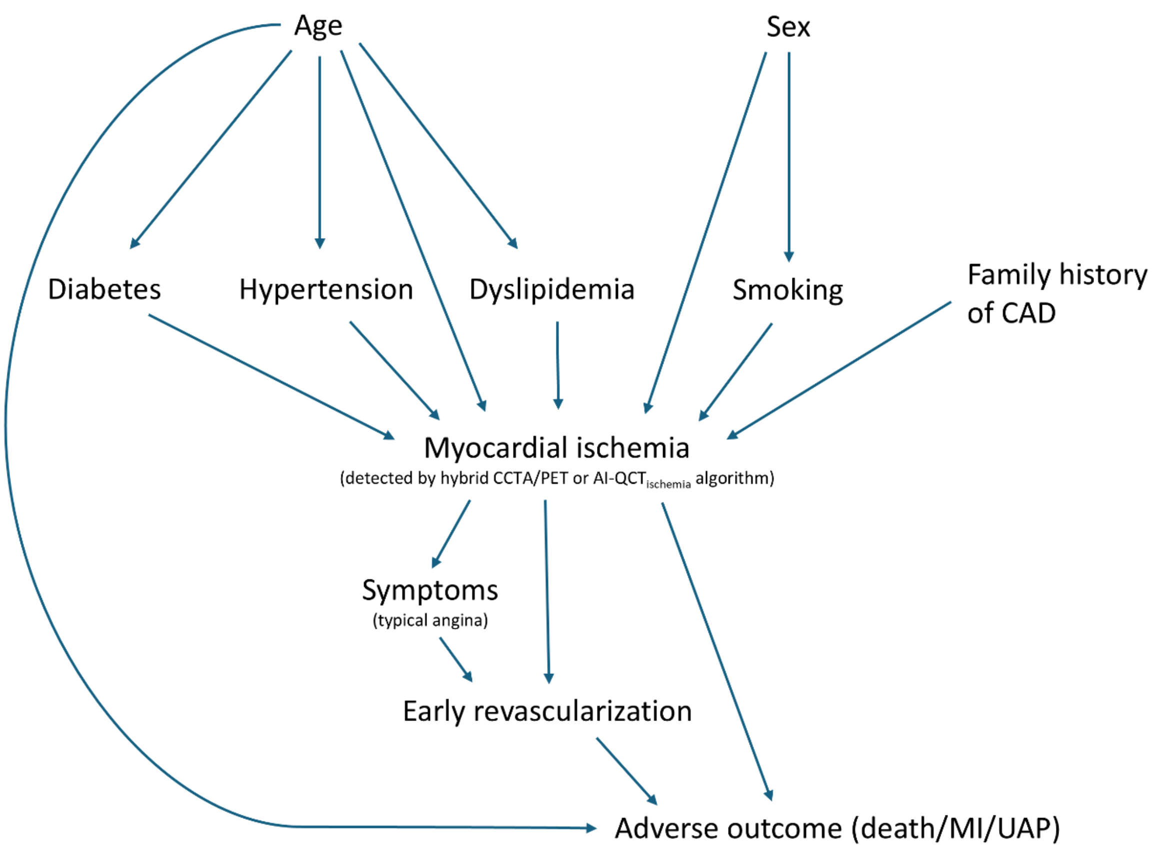

AI-QCT = artificial intelligence -guided quantitative computed tomography, CAD = coronary artery disease, CCTA = coronary computed tomography angiography, MI = myocardial infarction, PET = positron emission tomography, UAP = unstable angina pectoris.

Figure S2. Distribution of imaging findings in full analysis set (intention-to-diagnose). The number (%) of patients is shown according to the absence (–) or presence (+) of ischemic CAD by hybrid CCTA/PET imaging and normal (–) or abnormal (+) result by AI-QCT$_{ischemia}$ algorithm. Patients with inconclusive imaging results were considered as abnormal (+) in intention-to-diagnose approach. The annual rate of the composite endpoint (death/MI/UAP) with 95% confidence intervals is shown for each subgroup.

Full analysis set (n=2271)		AI-QCT$_{ischemia}$ algorithm	
		–	+
Hybrid CCTA/PET imaging	–	n=1263 (56%) 0.8 (0.7–1.0) %/year	n=506 (22%) 1.8 (1.4–2.3) %/year
	+	n=108 (5%) 3.0 (2.0–4.5) %/year	n=394 (17%) 3.4 (2.8–4.2) %/year

AI-QCT = artificial intelligence -guided quantitative computed tomography, CAD = coronary artery disease, CCTA = coronary computed tomography angiography, MI = myocardial infarction, PET = positron emission tomography, UAP = unstable angina pectoris.

Figure S3. Risk stratification by imaging findings in full analysis set (intention-to-diagnose). Kaplan–Meier survival curves (with shaded 95% confidence intervals) demonstrating the discriminative power of hybrid CCTA/PET imaging and AI-QCT$_{ischemia}$ algorithm in predicting long-term outcome (composite of all-cause mortality, myocardial infarction, and unstable angina pectoris).

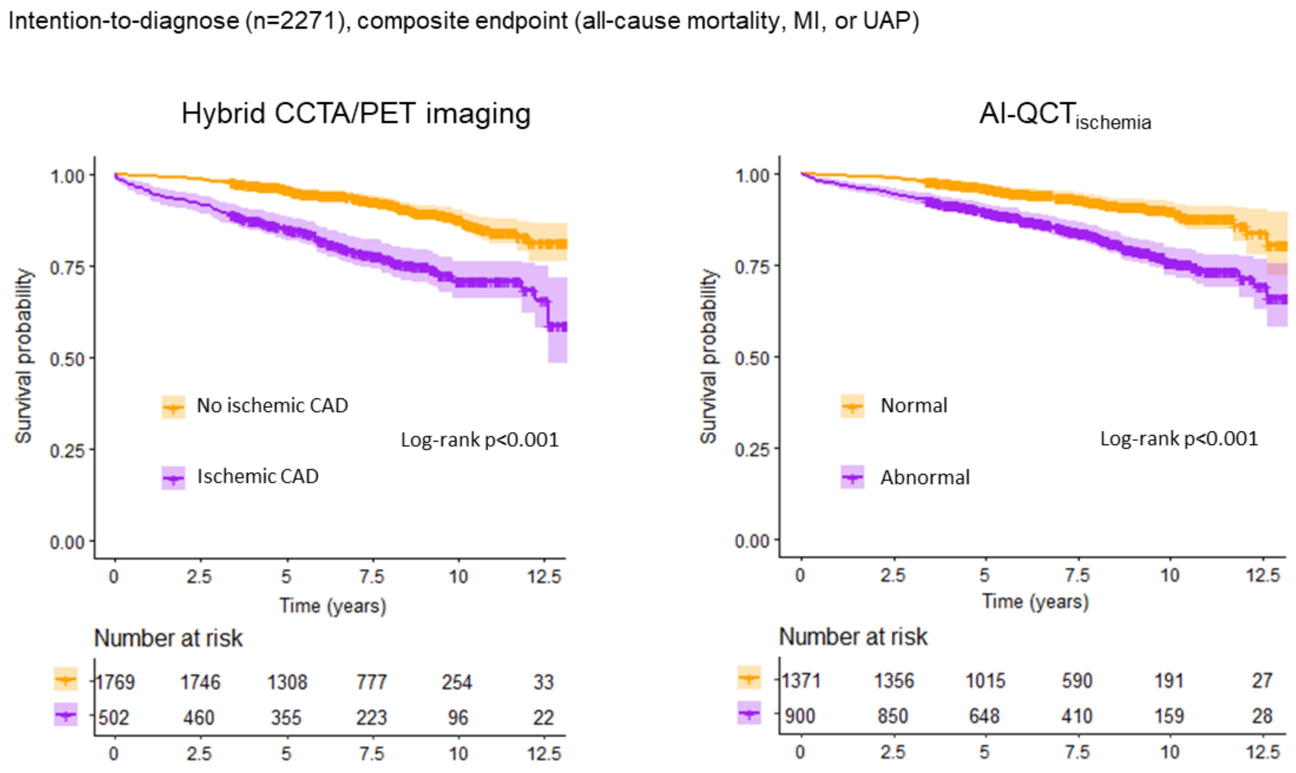

AI-QCT = artificial intelligence -guided quantitative computed tomography, CAD = coronary artery disease, CCTA = coronary computed tomography angiography, MI = myocardial infarction, PET = positron emission tomography, UAP = unstable angina pectoris.

Figure S4. Risk stratification for different event types in per-protocol set. Kaplan–Meier survival curves (with shaded 95% confidence intervals) demonstrating the discriminative power of hybrid CCTA/PET imaging and AI-QCT$_{ischemia}$ algorithm for predicting different event types in long-term follow-up. Abbreviations as in Supplemental Figure 2.

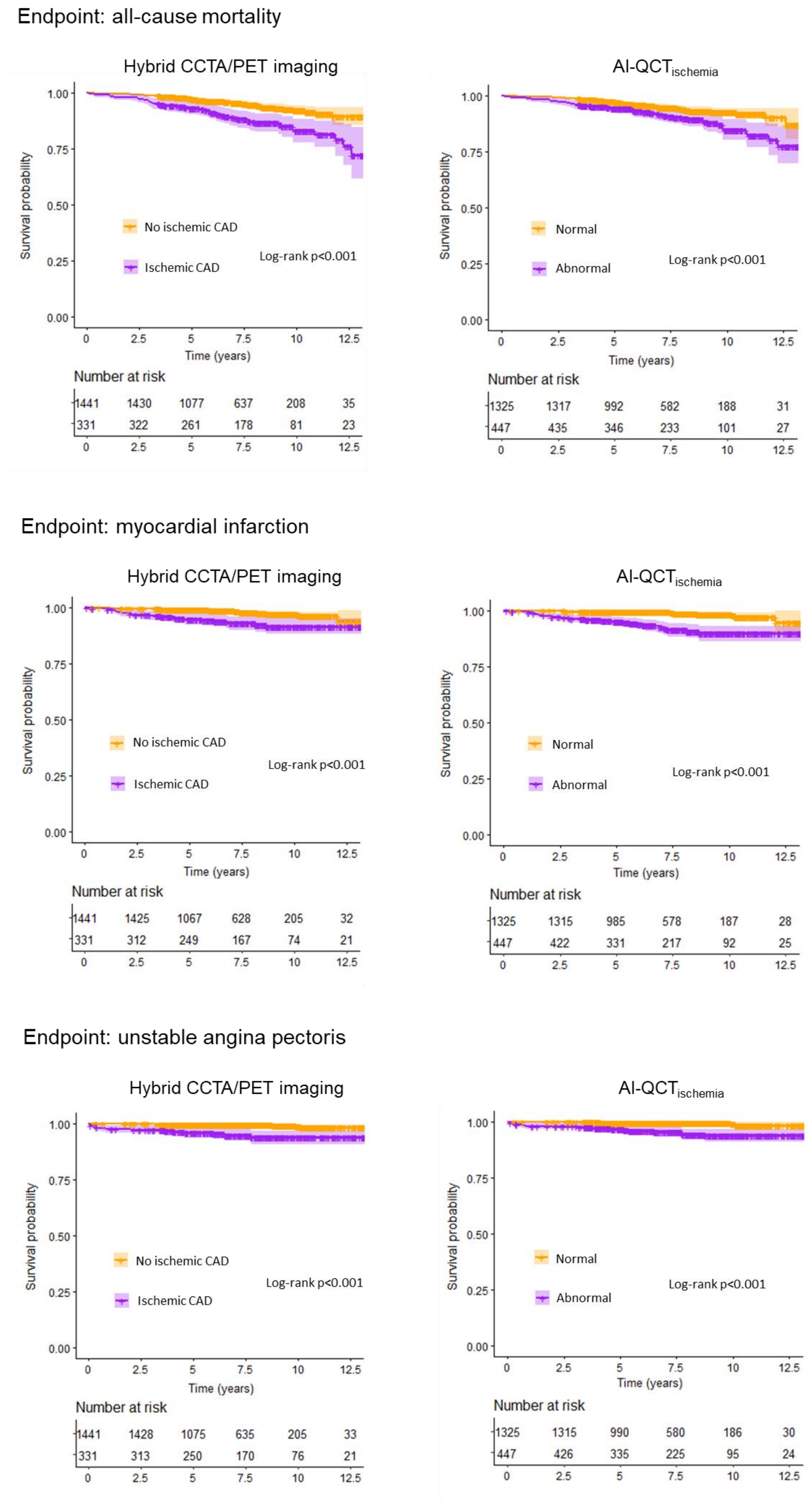

Figure S5. Predictive power of imaging findings in full analysis set (intention-to-diagnose). Receiver operating characteristic (ROC) curves are presented for different multivariable models in predicting the composite endpoint (death/MI/UAP). Harrell's C-index with 95% confidence interval is presented for different multivariable models.

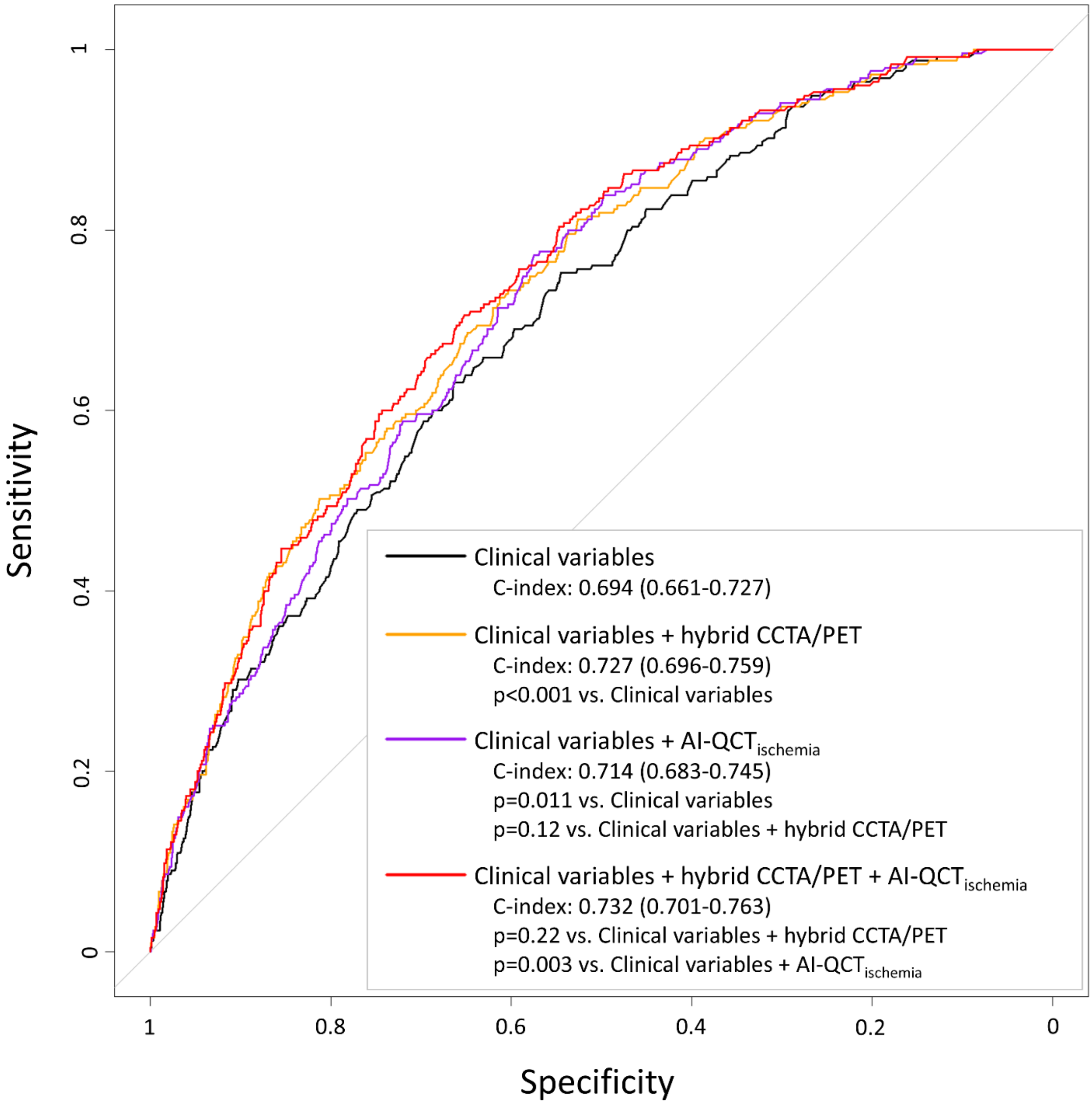

AI-QCT = artificial intelligence -guided quantitative computed tomography, CCTA = coronary computed tomography angiography, MI = myocardial infarction, PET = positron emission tomography, UAP = unstable angina pectoris.
